# Safety and clinical efficacy of an anti-PD-L1 antibody (c4G12) in dogs with advanced malignant tumours

**DOI:** 10.1371/journal.pone.0291727

**Published:** 2023-10-04

**Authors:** Naoya Maekawa, Satoru Konnai, Kenji Hosoya, Sangho Kim, Ryohei Kinoshita, Tatsuya Deguchi, Ryo Owaki, Yurika Tachibana, Madoka Yokokawa, Hiroto Takeuchi, Yumiko Kagawa, Satoshi Takagi, Hiroshi Ohta, Yukinari Kato, Satoshi Yamamoto, Keiichi Yamamoto, Yasuhiko Suzuki, Tomohiro Okagawa, Shiro Murata, Kazuhiko Ohashi

**Affiliations:** 1 Faculty of Veterinary Medicine, Department of Advanced Pharmaceutics, Hokkaido University, Sapporo, Japan; 2 Cancer Research Unit, One Health Research Center, Hokkaido University, Sapporo, Japan; 3 Faculty of Veterinary Medicine, Department of Disease Control, Hokkaido University, Sapporo, Japan; 4 Institute for Vaccine Research and Development (HU-IVReD), Hokkaido University, Sapporo, Japan; 5 Faculty of Veterinary Medicine, Veterinary Teaching Hospital, Hokkaido University, Sapporo, Japan; 6 Department of Companion Animal Clinical Sciences, Companion Animal Internal Medicine, School of Veterinary Medicine, Rakuno Gakuen University, Ebetsu, Japan; 7 North Lab, Sapporo, Japan; 8 Department of Veterinary Surgery 1, School of Veterinary Medicine, Azabu University, Sagamihara, Japan; 9 Department of Antibody Drug Development, Tohoku University Graduate School of Medicine, Sendai, Japan; 10 Department of Molecular Pharmacology, Tohoku University Graduate School of Medicine, Sendai, Japan; 11 Fuso Pharmaceutical Industries, Ltd., Osaka, Japan; 12 International Institute for Zoonosis Control, Hokkaido University, Sapporo, Japan; 13 Global Station for Zoonosis Control, Global Institution for Collaborative Research and Education (GI-CoRE), Hokkaido University, Sapporo, Japan; 14 Faculty of Veterinary Medicine, International Affairs Office, Hokkaido University, Sapporo, Japan; IRCCS Giovanni Paolo II Cancer Hospital, ITALY

## Abstract

Immune checkpoint inhibitors (ICIs) have been developed for canine tumour treatment, and pilot clinical studies have demonstrated their antitumour efficacy in dogs with oral malignant melanoma (OMM). Although ICIs have been approved for various human malignancies, their clinical benefits in other tumour types remain to be elucidated in dogs. Here, we conducted a clinical study of c4G12, a canine chimeric anti-PD-L1 antibody, to assess its safety and efficacy in dogs with various advanced malignant tumours (*n* = 12) at the Veterinary Teaching Hospital of Hokkaido University from 2018 to 2023. Dogs with digit or foot pad malignant melanoma (*n* = 4), osteosarcoma (*n* = 2), hemangiosarcoma (*n* = 1), transitional cell carcinoma (*n* = 1), nasal adenocarcinoma (*n* = 1), B-cell lymphoma (*n* = 1), or undifferentiated sarcoma (*n* = 2) were treated with 2 or 5 mg/kg c4G12 every 2 weeks. Treatment-related adverse events of any grade were observed in eight dogs (66.7%), including elevated aspartate aminotransferase (grade 3) in one dog (8.3%) and thrombocytopenia (grade 4) in another dog (8.3%). Among dogs with target disease at baseline (*n* = 8), as defined by the response evaluation criteria for solid tumours in dogs (cRECIST), one dog with nasal adenocarcinoma and another with osteosarcoma experienced a partial response (PR), with an objective response rate of 25.0% (2 PR out of 8 dogs; 95% confidence interval: 3.2–65.1%). These results suggest that c4G12 is safe and tolerable and shows antitumor effects in dogs with malignant tumours other than OMM. Further clinical studies are warranted to identify the tumour types that are most likely to benefit from c4G12 treatment.

## Introduction

Spontaneous tumours represent a major challenge in modern veterinary medicine, accounting for up to approximately 30% of death in dogs [1–3]. The most common malignancies in dogs include adenocarcinoma, mast cell tumour, lymphoma, and mammary tumour [4,5]. Current treatment options for canine tumours include surgery, radiotherapy, and conventional (cytotoxic) chemotherapy, and some molecular-targeted drugs have become available in veterinary clinics [6]. Despite recent advances in veterinary oncology, complete remission of the tumour is difficult to achieve in many cases; thus, the development of novel treatments, including immunotherapy, is urgently required.

Immune checkpoint inhibitors (ICIs), such as anti-programmed cell death 1 (PD-1) and anti-PD-ligand1 (PD-L1) antibodies, have been approved for the treatment of various tumour types in humans, including malignant melanoma, non-small cell lung cancer (NSCLC), and renal cell cancer [7–10]. Anti-PD-1/PD-L1 antibodies interrupt the immunosuppressive pathway induced by the interaction between the T cell coinhibitory receptor PD-1 and its ligand PD-L1, the latter of which is expressed in various cell types including immune and tumour cells [11–14]. ICIs have become a novel standard-of-care across multiple tumour types, and the focus of recent research has shifted to exploring biomarkers of response to ICIs, identifying primary and acquired mechanisms of resistance to ICI monotherapy, and developing ICI-based combination therapies [15–18]. Recently, anti-PD-1/PD-L1 antibodies were developed for canine tumour treatment, and their safety and antitumour efficacy were demonstrated in veterinary clinical pilot studies [19–22]. Although their clinical benefits have been strongly suggested in dogs with oral malignant melanoma (OMM), one of the most aggressive malignancies relatively common in dogs [23], it remains to be elucidated whether ICIs are effective in treating other tumour types in dogs.

PD-L1 immunohistochemistry of tumour tissues are used to select eligible patients for human ICI therapies in several clinical settings for specific cancer types [10,14]. In dogs, PD-L1 expression is reported in various tumour types including malignant melanoma, osteosarcoma, hemangiosarcoma, transitional cell carcinoma, nasal adenocarcinoma, and B-cell lymphoma [21,24–30], suggesting that these tumour types are potential target for canine ICI therapies. We have previously established a canine chimeric anti-PD-L1 antibody, c4G12, for canine cancer treatment. c4G12 efficiently binds to canine PD-L1 and blocks its interaction with PD-1, enhancing cytokine production and T cell proliferation in canine immune cell cultures [19]. The clinical efficacy of c4G12 was tested in canine OMM (*n* = 7) and undifferentiated sarcoma (*n* = 2). One dog with OMM and another with undifferentiated sarcoma showed clear antitumour responses (2/9 dogs, 22.2%) [19], demonstrating that immune checkpoint inhibition could be a novel option for the treatment of malignant tumours in dogs. The clinical study was expanded to examine its therapeutic potential in advanced, pulmonary metastatic OMM (*n* = 29); tumour regression was evident by diagnostic imaging in five dogs (17.2%), and a survival benefit was strongly suggested in comparison to a historical control group treated in the same veterinary hospital (median survival of 143 days vs. 54 days) [21]. Although some treatment-related adverse events (TRAEs) with potential immune-related causes (pneumonia and thrombocytopenia) were observed, c4G12 treatment was well tolerated and its safety profile was considered acceptable [21].

To facilitate the development of ICIs for canine tumours, in this study, we evaluated the safety and clinical efficacy of c4G12 in various types of advanced canine tumours at the Veterinary Teaching Hospital of Hokkaido University, Japan, from 2018 to 2023. We enrolled dogs (*n* = 12) with recurrent, metastatic, or resistant tumours (e.g., digit or foot pad malignant melanoma, osteosarcoma, and undifferentiated sarcoma) after at least one prior therapy, including surgery, radiation, or chemotherapy. TRAEs and antitumour responses were examined during c4G12 treatment given every 2 weeks.

## Materials and methods

### Overview of the clinical study

The clinical study using c4G12 was conducted with the approval of the Institutional Animal Care Committee of Hokkaido University (approval number: 15–0149 and 20–0041). The use of animals in the clinical study was approved by the Ethics Committee of the Faculty of Veterinary Medicine, Hokkaido University. Prior to enrolment in the clinical study, written informed consent was obtained from dog owners. Dogs with advanced malignant tumours (other than OMM) that were observed at the Hokkaido University Veterinary Teaching Hospital (HUVTH) between July 2018 and April 2023 were carefully screened for enrolment. Dogs with severe systemic illnesses unrelated to the tumour or concurrent tumours of different origins were excluded from the study. When a biopsy sample of the primary tumour (at any time point) was available for immunohistochemistry (IHC), PD-L1 expression in the tumour cells was examined at a commercial pathology laboratory (North Lab, Sapporo, Japan), as described previously [21]. c4G12 was administered intravenously to dogs at 2 or 5 mg/kg over 1 h using a syringe pump every 2 weeks until complete response, withdrawal of consent, death, or discontinuation due to unacceptable TRAEs, tumour progression, or deterioration of general conditions. No dogs were euthanised during the clinical study.

### Safety assessment

Physical examination, complete blood count, and blood chemistry were routinely performed at least every 6 weeks during treatment to monitor adverse events. Additional assessments including urinalysis, thoracic or abdominal radiography, ultrasonography, computed tomography (CT), and magnetic resonance imaging were performed when clinically required. Adverse events were classified and graded according to the Veterinary Cooperative Oncology Group–Common Terminology Criteria for Adverse Events (VCOG-CTCAE) v1.1 [31].

### Response evaluation

Tumour response to c4G12 treatment was defined according to the response evaluation criteria for solid tumours in dogs (cRECIST) v1.0 [32]. Baseline assessments were performed within 5 weeks prior to the first c4G12 dose. During the treatment, tumours were evaluated at least every 6 weeks using the same modality as the baseline assessment. The measurement modalities included clinical examination using callipers and diagnostic imaging using thoracic radiography, ultrasonography, or CT. When clinical or ultrasonographic examination was used for evaluation, special care was taken to minimise measurement error; the same investigator performed the re-evaluation using the same equipment as far as possible. Dogs with measurable, target lesion(s) (i.e., ≥10 mm on CT or clinical examination; ≥20 mm on thoracic radiograph or ultrasonographic examination) at baseline (*n* = 8) were considered “with target disease” and included in the response evaluation. Dogs with only non-measurable lesion(s) (i.e., <10 mm on CT or clinical examination; <20 mm on thoracic radiograph or ultrasonographic examination) at baseline (*n* = 4) were considered “with non-target disease”; however, tumour size was recorded as far as possible. Tumour response was defined as complete response (CR) if all detectable tumours disappeared, partial response (PR) if the reduction of the tumour diameter was ≥30%, progressive disease (PD) if the tumour increased by ≥20% or new lesion(s) appeared. If the tumour size was stable (decreased by less than 30% or increased by less than 20%) for at least six weeks, the tumour response was defined as stable disease (SD). Overall survival (in days) of the dogs was defined as the time from the first c4G12 dose to death. Dogs that were still alive at the time of writing the manuscript were recorded as censored data.

## Results

### Baseline characteristic of dogs and c4G12 treatment

In total, 12 dogs with advanced malignant tumours were enrolled in this study, including four with malignant melanoma (2 digit and 2 foot pad), two with osteosarcoma, two with undifferentiated sarcoma, and one each of the following tumour types: hemangiosarcoma, transitional cell carcinoma, nasal adenocarcinoma, and B-cell lymphoma. Three miniature schnauzers and one each of the following breeds: Chihuahua, miniature dachshund, Pomeranian, beagle, Shetland sheepdog, Siberian husky, Labrador retriever, Airedale terrier, and a mixed breed were included in the study. The median age at the time of enrolment was 12 years, ranging from 8 to 14 years. Four were male and eight were female, all of whom had been neutered before study enrolment. PD-L1 IHC using primary tumour tissue was performed in seven dogs, of which 5 were positive for PD-L1 expression. All dogs had received at least one prior therapy, including palliative or definitive surgery, radiation, and chemotherapy, and 10 dogs had metastatic disease at baseline. In 8 dogs, measurable target lesions were present at baseline assessment for tumour response evaluation by cRECIST [32] (“with target disease”), and the other 4 dogs lacked measurable lesions and were considered “with non-target disease” (Tables 1 and S1).

**Table 1:** Characteristics of dogs at baseline (*n* = 12).

Characteristic	
Tumour type―no. (%)	
Malignant melanoma	4 (33.3)
Digit	2 (16.7)
Foot pad	2 (16.7)
Osteosarcoma	2 (16.7)
Hemangiosarcoma	1 (8.3)
Transitional cell carcinoma	1 (8.3)
Nasal adenocarcinoma	1 (8.3)
B-cell lymphoma	1 (8.3)
Undifferentiated sarcoma	2 (16.7)
Age―year	
Median	12
Range	8–14
Age category―no. (%)	
≥10 year	9 (75.0)
<10 year	3 (25.0)
Sex―no. (%)	
Male	
Intact	0 (0)
Castrated	4 (33.3)
Female	
Intact	0 (0)
Spayed	8 (66.7)
PD-L1 status―no. (%)	
Positive	5 (41.7)
Negative	2 (16.7)
Not tested	5 (41.7)
Prior therapy―no. (%)	
Surgery	10 (83.3)
Radiation	4 (33.3)
Chemotherapy	8 (66.7)
Measurable lesion(s)―no. (%)	
Present	8 (66.7)
Absent	4 (33.3)

Dogs received intravenous c4G12 administration at 2 or 5 mg/kg every 2 weeks, with a median number of c4G12 doses of 4.5 times (range: 1–51 times) and a median treatment duration of 93 days (range: 14–739 days) (S2 Table). Dog #3 (foot pad malignant melanoma) received hypofractionated radiation therapy (4 fractions of 6.5 Gy, 1-week intervals, 26 Gy in total) from day 14 of c4G12 treatment to achieve local tumour control; thus, the local tumour was excluded from the response evaluation for c4G12 treatment. Dog #8 (transitional cell carcinoma) received concomitant chemotherapy with carboplatin (6–6.5 mg/kg i.v., every 3–4 weeks) from day 450 as a combination therapy with c4G12 treatment to achieve better tumour control.

### Adverse events related to c4G12 treatment

TRAEs of any grade were observed in 8 dogs (66.7%), including 2 TRAEs of grade 3 or higher (16.7%) (Table 2). Common TRAEs that occurred in at least two dogs included increased levels of alkaline phosphatase, alanine aminotransferase (ALT), aspartate aminotransferase (AST), and creatinine, and anorexia. One dog (#10, B-cell lymphoma) experienced a grade 3 increase in AST levels; however, this was not accompanied by any clinical symptoms. Neither c4G12 treatment discontinuation nor additional medication was required. Another dog (#11, undifferentiated sarcoma) developed grade 4 thrombocytopenia (19,500/μL) 9 days after the first c4G12 administration. This might be attributable to immune-related thrombocytopenia induced by c4G12 treatment, although the platelet count was considerably low (33,000/μL) at baseline (before c4G12 treatment) due to the prior chemotherapy with metronomic chlorambucil (8.4 mg/m^2^ p.o., q48 h) which was stopped 24 days before the first c4G12 administration. The c4G12 treatment was stopped and the dog was treated with glucocorticoid (prednisolone), after which the platelet count recovered (70,500/μL) on day 18. In addition, another dog (#4, food pad malignant melanoma) developed an allergic reaction (sudden vomiting and decrease in general activity; grade 2) immediately after the second c4G12 administration which resolved soon after the administration of antihistamine drugs. c4G12 treatment was discontinued for this dog. No unusual adverse events were observed when c4G12 was used concomitantly with hypofractionated radiotherapy (dog #3) or carboplatin chemotherapy (dog #8).

**Table 2:** Treatment-related adverse events (TRAEs) (*n* = 12).

TRAEs―no. (%)	Any grade	Grade 3 or 4	Leading to discontinuation
Any TRAEs	8 (66.7)	2 (16.7)	2 (16.7)
Alkaline phosphatase	4 (33.3)	0 (0)	0 (0)
Allergic reaction/hypersensitivity	1 (8.3)	0 (0)	1 (8.3)
Alopecia	1 (8.3)	0 (0)	0 (0)
ALT	3 (25.0)	0 (0)	0 (0)
Anorexia	2 (16.7)	0 (0)	0 (0)
AST	1 (8.3)	1 (8.3)	0 (0)
Creatinine	2 (16.7)	0 (0)	0 (0)
Diarrhoea	1 (8.3)	0 (0)	0 (0)
Lethargy/fatigue/general performance	1 (8.3)	0 (0)	0 (0)
Lipase	1 (8.3)	0 (0)	0 (0)
Thrombocytopenia	0 (0)	1 (8.3)	1 (8.3)
Vomiting	1 (8.3)	0 (0)	0 (0)

ALT, alanine aminotransferase; AST, aspartate aminotransferase.

Grading was performed according to the VCOG-CTCAE v1.1 [31].

### Tumour response to c4G12 treatment

**Fig 1:**
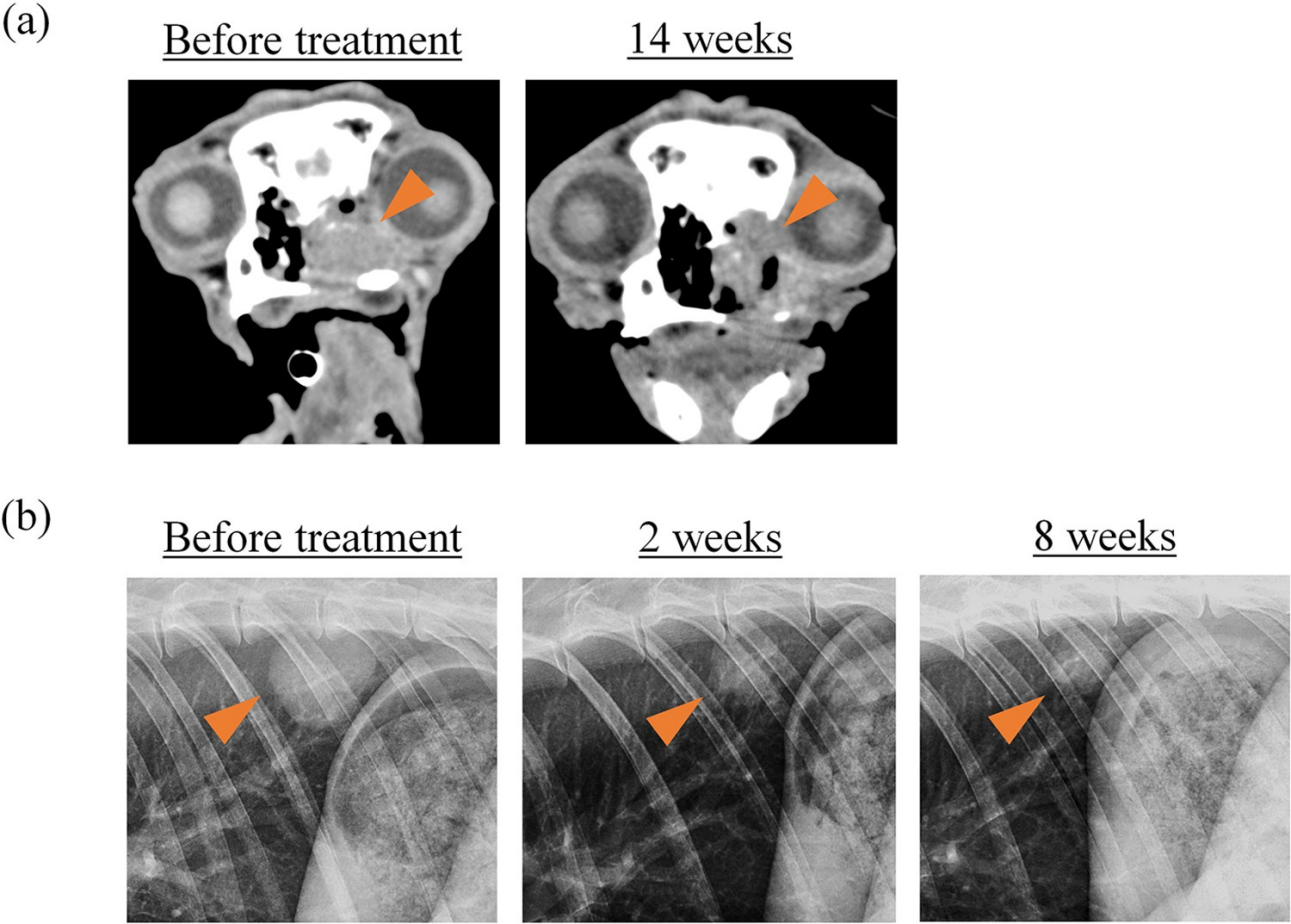
Tumour response to c4G12 treatment in dogs with target disease. (a) Nasal adenocarcinoma in the nasal cavity (dog #9, 18 mm at baseline, depicted by CT) responded to c4G12 treatment at week 14 (12 mm, PR). (b) Metastatic osteosarcoma in the lung (dog #6, 33 mm at baseline, depicted by thoracic radiography) responded to c4G12 treatment at week 2 (19 mm, PR). The response lasted for at least 8 weeks (21 mm).

Among the 12 dogs treated in this study, eight had the target disease and were eligible for response evaluation by cRECIST [32]. Among them, three were considered “not evaluable (NE)” because c4G12 treatment was discontinued due to TRAEs (dog #11) or deterioration of general conditions (#10 and #12) before the first re-evaluation of the tumour. Other three dogs (#3, #4, and #7) were considered PD on days 35, 14, and 14, respectively. In one dog with nasal adenocarcinoma (#9), a recurrent tumour which had progressed after prior radiation therapy responded to c4G12 treatment on day 98 (14 weeks). The longest tumour diameter decreased by 33.3% (from 18 to 12 mm; PR) (Fig 1A). The dog died on day 173 of treatment for unspecified reasons (decline in the general condition) that were not attributable to tumour progression or TRAEs. In another dog with osteosarcoma (#6), a metastatic lesion in the lung, that emerged during adjuvant chemotherapy with carboplatin after amputation and progressed during subsequent doxorubicin and toceranib treatment, clearly responded to c4G12 treatment on day 14. The longest tumour diameter decreased by 42.4% (from 33 to 19 mm; PR) (Fig 1B). The response was durable and lasted for at least 8 weeks (21 mm on day 56), and the dog was still on treatment at the time of writing. The objective response rate (ORR) was 25.0% (2 PR out of 8 dogs) with a 95% confidence interval of 3.2–65.1% (Table 3).

**Table 3:** Tumour response to c4G12 treatment in dogs with target disease (*n* = 8).

Best overall response―no. (%)	
CR	0 (0)
PR	2 (25.0)
SD	0 (0)
PD	3 (37.5)
NE	3 (37.5)
ORR―% (95% CI)	25.0 (3.2–65.1)

CR, complete response; PR, partial response; SD, stable disease; PD, progressive disease; NE, not evaluable.

ORR, objective response rate (CR+PR); CI, confidence interval.

**Fig 2:**
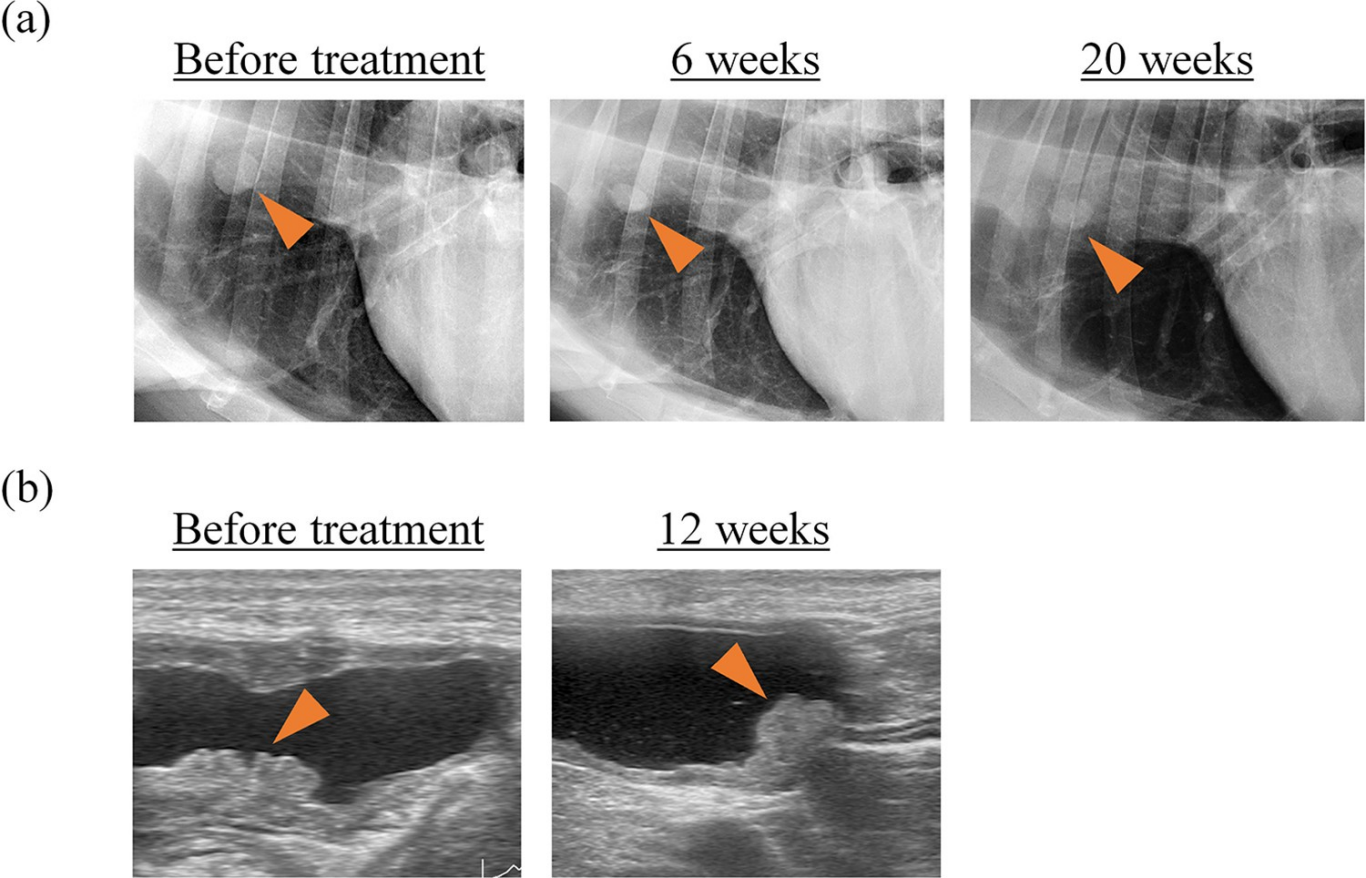
Tumour response to c4G12 treatment in dogs with non-target disease. (a) Metastatic digit malignant melanoma in the lung (dog #2, 15 mm at baseline, depicted by thoracic radiography) was stable at week 6 on c4G12 treatment (14 mm). The response lasted for at least 20 weeks (16 mm). (b) Transitional cell carcinoma in the bladder (dog #8, 8 mm at baseline, depicted by ultrasonography) was stable at week 12 on c4G12 treatment (9 mm).

In the four dogs with non-target disease, unequivocal progression was noted in two dogs (#1 and #5) on days 40 and 42, respectively. In one dog with digit malignant melanoma (#2), a non-measurable metastatic lung lesion depicted on thoracic radiography (15 mm) appeared stable on day 42 (6 weeks, 14 mm) (Fig 2A). The response lasted for at least 20 weeks (16 mm on day 140), and the dog was still undergoing treatment at the time of writing this report. In another dog with non-measurable transitional cell carcinoma (#8), the bladder tumour depicted on ultrasonography (8 mm) progressed very slowly with c4G12 treatment and was considered stable on day 86 (12 weeks, 9 mm) (Fig 2B). The response was durable and persisted until unequivocal progression was confirmed on day 289. Although the dog received carboplatin chemotherapy in combination with c4G12 from day 450, the tumour continued to progress slowly, and the dog died on day 739.

## Discussion

Given the broad indications of human ICIs and the expression of PD-L1 in canine tumours, ICIs may also be beneficial across canine tumour types. However, the safety and efficacy of ICIs in canine tumours other than OMM are currently unclear, limiting the potential use of emerging ICIs for the treatment of multiple tumours. The aim of this study was to provide a clue for the expansion of the use of ICIs in canine tumours. Indeed, treatment with c4G12 was well tolerated in these 12 dogs, with a low frequency of treatment discontinuation due to TRAEs. Evidence of clinical antitumour response has been reported in dogs with nasal adenocarcinoma and osteosarcoma. In addition, the clinical benefit of c4G12 treatment has been strongly suggested in dogs with digit malignant melanoma and transitional cell carcinoma, although the evaluation of tumour response in these dogs was based solely on non-measurable lesions; thus, the results should be interpreted with caution. Nonetheless, to identify canine tumour types that could be treated with c4G12, further clinical studies involving a larger number of dogs with each tumour type should be performed, particularly in dogs with nasal adenocarcinoma, osteosarcoma, digit malignant melanoma, and transitional cell carcinoma.

The TRAEs observed in this study suggested possible immune-related side effects of c4G12, including hepatic, renal, gastrointestinal, and pancreatic toxicities. The exacerbation of thrombocytopenia that developed under prior metronomic chlorambucil (commonly reported in dog clinical studies [33–35]) in a dog with undifferentiated sarcoma (#11) was also possibly immune-related because the platelet count recovered soon after immunosuppressant treatment. Although the frequency is considered rare, immune thrombocytopenia has also been reported in ICI therapies for human malignancies [36,37]. While the detailed mechanism underlying this observation is unclear, careful consideration is needed when selecting dogs for the safer use of c4G12; dogs with active immune-related disorders, including autoimmune diseases, or those with such a history should be included with special attention. The type, severity, and frequency of TRAEs were consistent with those reported for ICIs in dogs [19–22] and humans [7,8], suggesting that the safety profile is acceptable for treating dogs with advanced tumours. Because cutaneous, endocrine, pulmonary, and nervous system toxicities have also been reported for human ICIs [38,39], veterinarians must be well prepared for possible side effects when using ICIs in dogs.

To the best of our knowledge, this is the first report to show that ICIs are potentially applicable to the treatment of canine nasal adenocarcinoma, osteosarcoma, digit malignant melanoma, and transitional cell carcinoma. The clinical activity of c4G12 suggests that the PD-1/PD-L1 axis is an important immune evasion mechanism in various canine tumour types, as has been reported in humans. In human medicine, ICIs are available for the treatment of a wide variety of cancer types including malignant melanoma, NSCLC, renal cell cancer, squamous cell carcinoma, Hodgkin’s lymphoma, urothelial carcinoma, and colorectal cancer [9,10], and their indications are rapidly expanding to other cancer types. Immune landscape of canine tumours remains largely unknown, however, recent studies on canine osteosarcoma and bladder carcinoma revealed that manipulation of immune cell subsets can induce antitumor efficacies in specific clinical settings [40–43]. Considering the importance of antitumour immunity in tumour control and the high positive rate of PD-L1 expression [21], immune checkpoint blockade using c4G12 may be an attractive treatment option for these tumour types. In this study, however, the clinical implications of PD-L1 expression in tumour biopsies remained unclear because the dog with PD-L1–negative nasal adenocarcinoma (#9) responded clearly to c4G12 treatment. The biopsy sample was obtained 11 months before the initiation of immunotherapy, and during which the dog underwent radiotherapy which may have altered the immunological microenvironment inside the tumour [44]. Because PD-L1 expression in tumour tissues is inducible via various mechanisms at the genetic, epigenetic, transcriptional, post-transcriptional, and post-translational levels [45–47], the timing of biopsy for PD-L1 IHC is important for assessing PD-L1 expression as a biomarker of response to ICI therapies. Although little is known about the molecular mechanisms regulating PD-L1 expression in dogs, our recent studies revealed the transcriptional [48] and post-translational [49] control of PD-L1 in canine tumour cells. A better understanding of the kinetics of PD-L1 expression would help establish a good biomarker for canine ICIs and provide clues to elucidate the mechanisms of resistance.

Limitations of this study include the small sample size for each tumour type and the lack of genetic and immunological analysis of the tumour tissues. Because ORRs are generally low in patients treated with ICI monotherapy [9], reliable ORRs for each canine tumour type should be calculated in future clinical studies with larger populations of dogs. Biomarker analyses using tumour tissues such as tumour mutational burden, microsatellite instability/mismatch repair deficiency, or PD-L1 scoring in tumour and immune cells [15] may provide a strong rationale to select eligible dogs that can be successfully treated with ICIs.

In conclusion, this clinical study demonstrates the potential of c4G12 as a promising immunotherapeutic drug for advanced canine tumours in addition to OMM. Further studies are warranted to examine the detailed clinical benefits of c4G12 for each tumour type, including its safety profile, ORR, and overall survival in specific disease conditions (e.g., stage, prior therapies, and gene alterations). Given their great success in humans, ICIs would become a powerful therapeutic option for various canine tumours as monotherapy or combination therapy with existing treatment modalities such as surgery, radiation, conventional chemotherapy, molecular-targeted drugs, and upcoming immunotherapy. The results from canine clinical studies using ICIs may be extrapolated to human cancer studies as a large animal model of cancer in which the tumour develops spontaneously in an immunocompetent host and resembles its human counterpart in biological behaviour and response/resistance to treatment.

## Supporting information

S1 Table Characteristics of dogs enrolled in clinical study. (PDF)

S2 Table Summary of c4G12 treatment. (PDF)
