# Supplementary material for: Safety and clinical efficacy of an anti-PD-L1 antibody (c4G12) in dogs with advanced malignant tumours
Source: PLoS One. 2023 Oct 4;18(10):e0291727. doi: 10.1371/journal.pone.0291727 (PMC10550157; doi:10.1371/journal.pone.0291727)
Supplement: S1 Table — (PDF) [file pone.0291727.s001.pdf]

Supplementary Information for

Safety and clinical efficacy of an anti-PD-L1 antibody (c4G12) in dogs with advanced malignant tumours

Naoya Maekawa, Satoru Konnai*, Kenji Hosoya, Sangho Kim, Ryohei Kinoshita, Tatsuya Deguchi, Ryo Owaki, Yurika Tachibana, Madoka Yokokawa, Hiroto Takeuchi, Yumiko Kagawa, Satoshi Takagi, Hiroshi Ohta, Yukinari Kato, Satoshi Yamamoto, Keiichi Yamamoto, Yasuhiko Suzuki, Tomohiro Okagawa, Shiro Murata, Kazuhiko Ohashi

*Corresponding author email:
konnai@vetmed.hokudai.ac.jp

S1 Table. Characteristics of dogs enrolled in clinical study.

Dog #	Breed	Sex	Age (year)	Tumour type	Primary site	Metastatic site	PD-L1 expression	Prior therapy
1	Miniature schnauzer	Female, spayed	12	Malignant melanoma	Digit	Lymph node, skin, lung	ND	Surgery, chemotherapy
2	Airedale terrier	Male, castrated	9	Malignant melanoma	Digit	Lung	+	Surgery
3	Beagle	Female, spayed	13	Malignant melanoma	Foot pad	Lymph node, lung	ND	Surgery
4	Miniature schnauzer	Female, spayed	12	Malignant melanoma	Foot pad	Skin	-	Surgery
5	Pomeranian	Male, castrated	8	Osteosarcoma	Limb	Lung	ND	Surgery, chemotherapy
6	Siberian husky	Female, spayed	11	Osteosarcoma	Limb	Lung	+	Surgery, chemotherapy
7	Labrador retriever	Female, spayed	12	Hemangiosarcoma	Bone	Lung, liver, spleen, skin	+	Radiation, chemotherapy, surgery
8	Shetland sheepdog	Female, spayed	12	Transitional cell carcinoma	Bladder	None	ND	Radiation, chemotherapy
9	Mix	Male, castrated	14	Nasal adenocarcinoma	Nasal cavity	None	-	Radiation
10	Miniature schnauzer	Female, spayed	13	B-cell lymphoma	Lymph node	Lymph node	+	Surgery, chemotherapy

11	Chihuahua	Male, castrated	9	Undifferentiated sarcoma	Limb	Lymph node, lung, skin	+	Surgery, chemotherapy, radiation
12	Miniature dachshund	Female, spayed	11	Undifferentiated sarcoma	Small intestine	Lung, liver	ND	Surgery, chemotherapy

ND, not determined.
