# Supplementary material for: Safety and clinical efficacy of an anti-PD-L1 antibody (c4G12) in dogs with advanced malignant tumours
Source: PLoS One. 2023 Oct 4;18(10):e0291727. doi: 10.1371/journal.pone.0291727 (PMC10550157; doi:10.1371/journal.pone.0291727)
Supplement: S2 Table — (PDF) [file pone.0291727.s002.pdf]

S2 Table. Summary of c4G12 treatment.

Dog #	Number of c4G12 doses	Overall survival (day)	Amount of dosage	Concomitant therapy	Measurable lesion	BOR	Treatment-related adverse events (grade)
1	7	93†[a]	5 mg/kg	None	Absent	-	Alkaline phosphatase (1), ALT (2), AST (1), Vomiting (1)
2	11	140†[b]	2 mg/kg	None	Absent	-	Creatinine (1)
3	5	60	5 mg/kg	Radiation*	Present	PD	None
4	1	14†[a]	5 mg/kg	None	Present	PD	Allergic reaction/hypersensitivity (2)
5	32	445	5 mg/kg	None	Absent	-	Alopecia (1), Lipase (1)
6	4	56†[b]	2 mg/kg	None	Present	PR	None
7	1	14†[a]	2 mg/kg	None	Present	PD	None
8	51	739	5 mg/kg	Chemotherapy**	Absent	-	Creatinine (2)
9	13	173	5 mg/kg	None	Present	PR	None
10	1	21	5 mg/kg	None	Present	NE	Alkaline phosphatase (2), ALT (1), AST (3)
11	1	33	5 mg/kg	None	Present	NE	Alkaline phosphatase (2), ALT (1), Anorexia (1), Lethargy/fatigue/general performance (1), Thrombocytopenia (4)
12	1	21†[a]	5 mg/kg	None	Present	NE	Alkaline phosphatase (2), Anorexia (1), Diarrhoea (1)

BOR, best overall response; PD, progressive disease; PR, partial response; NE, not evaluable.

†Censored due to (a) loss to follow-up or (b) alive at the time of writing.

*The dog was treated with hypofractionated radiotherapy (four fractions of 6.5 Gy, once a week) from day 14 of c4G12 therapy.

**The dog was treated with carboplatin (6–6.5 mg/kg i.v., every 3–4 weeks) from day 450 of c4G12 treatment.
